# Determinants of child restraint system selection and installation practices: implications for injury prevention and public health implementation

**DOI:** 10.3389/fpubh.2026.1831898

**Published:** 2026-06-03

**Authors:** Marta Kopańska, Damian Frej, Małgorzata Wasacz

**Affiliations:** 1 Department of Medical Communication and Professional Competency Development Faculty of Medicine, Collegium Medicum, University of Rzeszów, Rzeszów, Poland; 2 Department of Automotive Engineering and Transport, Kielce University of Technology, Kielce, Poland

**Keywords:** caregiver behavior, child comfort, child restraint systems (CRS), child safety in the vehicle, CRS installation, ISOFIX, selection determinants

## Abstract

Road traffic injuries remain a leading cause of death and serious harm among children, and a substantial proportion of this burden occurs among child passengers. Despite clear safety recommendations, incorrect installation and suboptimal child restraint system (CRS) selection remain widespread, representing a persistent public health implementation gap. Against this backdrop, the present study examined determinants of CRS selection decisions and self-reported installation and use practices, with a particular focus on how selection and retail-support experiences relate to intention to choose the same CRS again. A cross-sectional online survey (Google Forms) was conducted from February to December 2024, yielding 3,750 questionnaires, of which 1,150 complete cases were included in analysis. Descriptive statistics were supplemented with chi-square tests (Cramér's V) and multivariable logistic regression (odds ratios, 95% CI) for top-box intention to choose the same CRS again (“yes/probably yes”). Price (58.1%), seat weight (48.2%), and installation method (46.1%) were the most frequently reported selection determinants, while safety tests (28.3%) and comfort (26.4%) were less commonly cited. ISOFIX solutions predominated (74.6%), with installation most often behind the driver (45.0%) and a notable share on the front seat (25.4%). Intention to choose the same CRS again was low (27.4% top-box; 62.3% negative). Seller installation demonstration was associated with intention to choose the same CRS again (χ^2^ = 22.82, *p* = 0.0036; V = 0.100), whereas reported child discomfort was not (*p* = 0.9378). In logistic regression, only age 26–35 years (vs. 18–25) predicted top-box intention to choose the same CRS again (OR = 1.42, 95% CI 1.05–1.91), with overall low explanatory power (McFadden pseudo-R^2^ = 0.0167). The findings suggest that economic and usability considerations outweigh explicitly stated safety criteria, and that closing the implementation gap requires stronger, scalable support for correct CRS selection and installation.

## 1 Introduction

Road traffic injuries remain a major cause of death and serious harm among children worldwide, and child passengers continue to represent an important part of the road safety burden in Europe despite long-term improvements in prevention and enforcement (1–4). For children traveling in private vehicles, effective injury prevention depends not only on the availability of CRS, but also on their consistent use, correct installation, and appropriate matching to the child's size and developmental stage (3, 5). Current clinical and public health guidance recommends a staged approach to protection, including prolonged rear-facing travel for younger children, followed by forward-facing restraint with a harness and, later, booster use, with transition decisions based primarily on height, weight, and manufacturer limits rather than age alone (5, 6). Although legislation and policy frameworks can improve child passenger protection, their effectiveness depends on how well safety recommendations are translated into routine caregiver practice (7, 8).

In practice, this translation remains incomplete. European monitoring data indicate that CRS use and related protective behaviors are still suboptimal in a proportion of journeys, and that correct CRS use remains a continuing public health concern (3, 4, 9). A major challenge is not only non-use, but also misuse. Studies have shown that caregivers frequently make errors such as loose installation, incorrect belt or anchor routing, and inadequate harness fit, all of which may reduce the protective effect of the CRS (10–12). Importantly, misuse has been observed both in CRS installed with the vehicle seat belt and in those using ISOFIX anchorage, suggesting that technical solutions alone do not eliminate the risk of error (11). This creates a persistent evidence-to-practice gap in which formal safety standards and recommendations do not reliably result in correct everyday CRS use.

For this reason, increasing attention has been directed toward implementation-oriented interventions designed to support caregivers in selecting and using CRS correctly. A systematic review of CRS interventions suggests that educational and behavioral strategies can improve correct use, particularly when practical guidance is included (13). Randomized and community-based studies have reported benefits from demonstration-based education, observational learning, and targeted safety programmes (14, 15). More recently, virtual and technology-supported approaches have been explored, including remote counseling, interactive virtual presence, and digital monitoring tools that may help caregivers maintain correct installation and use over time (16–21). These approaches are particularly relevant where access to in-person fitting support is limited.

At the same time, CRS-related decisions are shaped by more than technical knowledge alone. Previous studies indicate that caregiver behavior is influenced by sociodemographic characteristics, safety knowledge, behavioral skills, perceived convenience, and access to reliable information (22–24). Research also shows that information sources vary substantially across families and may affect both CRS selection and subsequent use practices (25, 26). In addition, real-world CRS choice often involves trade-offs between safety, ease of installation, usability, and product characteristics, which may influence how caregivers evaluate the CRS after purchase and whether they remain satisfied with it in daily use (27, 28). Against this background, locally grounded evidence is needed to better understand how caregivers choose CRS, what support they receive, and how these experiences relate to reported installation and use practices. Therefore, the present study aimed to examine determinants of CRS selection, reported installation and everyday use practices, and selected retail-support experiences among caregivers, with particular attention to factors associated with the intention to choose the same CRS again. This general aim was further operationalized through a set of specific research questions addressed in the Materials and Methods section.

## 2 Materials and methods

This section describes the study design, participant recruitment, data collection procedure, screening and quality control, variable operationalization, and statistical methods used in the analysis.

### 2.1 Study design and aims

This study was designed as a quantitative cross-sectional survey based on a standardized online questionnaire. The main aim of the study was to identify the determinants of CRS selection, to identify the sources of information used by caregivers, to describe declared installation and use practices, and to indicate areas of potential competence gap requiring implementation support. The analytical part of the study addressed four research questions. First, it was examined whether seller-provided installation demonstration was associated with intention to choose the same CRS again. Second, it was assessed whether declared child discomfort during travel, expressed for example as crying or fussiness in the CRS, was associated with lower intention to choose the same CRS again. Third, it was tested whether stronger safety orientation was associated with a higher proportion of positive responses regarding intention to choose the same CRS again. Fourth, a multivariable model was estimated to identify factors associated with a positive response while controlling simultaneously for selected sociodemographic, usage-related, and retail-support variables.

### 2.2 Participants, recruitment, and data collection procedure

The target population comprised drivers who traveled with at least one child aged 6 years or younger. Eligibility for participation required meeting two inclusion criteria: holding a valid driving license and traveling with a child not older than 6 years. Data were collected from February 2024 to December 2024 using a questionnaire prepared in Google Forms. The survey link was disseminated through multiple thematic groups on social media platforms, particularly Facebook, including groups focused on parenting, children, and child transport. Participation was voluntary, anonymous, and unpaid. The questionnaire began with two screening questions concerning possession of a driving license and traveling with a child aged 6 years or younger. These questions served as an eligibility check. If a respondent answered negatively to either question, the survey was terminated automatically and a message thanking the respondent for their interest in the study was displayed. Such entries were not included in the analytical dataset. The questionnaire included items on respondent characteristics, place of residence, number of children, travel frequency with a child, weekly CRS use time, type of CRS attachment, seat installation location in the vehicle, factors influencing CRS selection, seller-provided support at the time of purchase, sources of information used during seat selection, child discomfort during travel, and intention to choose the same CRS again. No directly identifying personal data were collected. Completion of the questionnaire was treated as provision of informed consent to participate in the study.

### 2.3 Screening, quality control, and analytical sample

A total of 3,750 survey entries were recorded, including entries terminated at the screening stage. Quality control was based on a two-stage procedure. First, entries from respondents who did not meet the eligibility criteria were excluded automatically on the basis of the two screening questions. Second, among eligible questionnaires, records with missing data in variables required for the descriptive, comparative, and multivariable analyses were excluded in accordance with a complete-case approach. The final analytical sample consisted of 1,150 complete questionnaires, which formed the basis for all reported distributions of counts and percentages and for all inferential analyses. This approach ensured internal consistency of the analytical dataset across the bivariate and multivariable models.

### 2.4 Variables and operationalization

The primary outcome variable was intention to choose the same CRS again. This variable was used as an indirect indicator of satisfaction with the currently used CRS rather than as a literal declaration of future repurchase of the identical model for the same child. As children grow, they may require a different CRS category or eventually transition to seat-belt use; therefore, this variable was interpreted as a summary indicator of caregiver satisfaction with the current CRS choice and experience. In practical terms, the question was intended to assess whether respondents were satisfied enough with their current purchase that they would choose the same CRS again under comparable circumstances.

For the purposes of association analyses and multivariable logistic regression, the variable was categorized into a positive “top-box” category, including the responses “yes” and “probably yes”, and a non-positive category, including all remaining responses.

The safety orientation indicator was defined as a synthetic score ranging from 0 to 4 points. One point was assigned for each of the following components: paying attention to safety certificates at the time of purchase, indicating safety tests as a CRS selection factor, using ISOFIX attachment, and reporting possession of a system supporting correct installation. Higher values therefore reflected stronger orientation toward explicitly safety-related CRS features and practices.

The sales support indicator was defined as a measure of the scope of seller-provided support during the purchase process. It was based on whether the seller demonstrated CRS installation and securing the child, offered an in-car fit check, and allowed the child to test the seat before purchase. Higher values represented broader retail support.

Additional variables included sociodemographic characteristics, number of children in the household, place of residence, travel frequency with a child, weekly time the child spent in the CRS, CRS attachment type, and seat installation location in the vehicle. Place of residence was categorized according to settlement size as rural area, town or city with fewer than 50,000 inhabitants, town or city with 50,000–150,000 inhabitants, town or city with 150,000–500,000 inhabitants, and town or city with more than 500,000 inhabitants.

Child discomfort during travel was treated as a usage-related variable reflecting caregiver-reported crying, whining, or fussiness in the CRS. For selected comparative analyses, child discomfort responses were grouped into three broader categories: frequent discomfort, occasional discomfort, and rare or no discomfort.

For the correlation analysis presented as a heat map, the selected questionnaire items included intention to choose the same CRS again, child crying or fussiness in the CRS, whether the seller demonstrated CRS installation and securing the child, whether the seller offered an in-car fit check, whether the child could test the CRS at purchase, attention to safety certificates, use of other users' advice or opinions, and weekly hours the child spent in the CRS. These variables were selected because they represented key domains of interest in the study, namely caregiver evaluation of the CRS, child travel experience, retail support during purchase, attention to safety-related information, social information sources, and routine exposure to CRS use.

### 2.5 Statistical analysis

The analyses were carried out in stages. First, descriptive statistics were used to characterize the sample and the distribution of responses in the key variables. Frequencies and percentages were reported for categorical variables. Next, cross-tabulations with within-group percentages and chi-square tests of independence were used to compare the distribution of responses between groups, and the strength of the associations was summarized using Cramér's V. Multivariable logistic regression was then applied to identify factors associated with a positive top-box response for intention to choose the same CRS again while controlling simultaneously for multiple predictors. The results were presented as odds ratios, 95% confidence intervals, and *p*-values. Model fit was described using McFadden's pseudo-R^2^ as well as the Akaike information criterion (AIC) and Bayesian information criterion (BIC).

In addition, Spearman rank correlations were calculated to provide a compact overview of co-occurrence patterns across selected questionnaire items and were presented in the form of a heat map. Statistical significance in the figure was marked symbolically at three levels corresponding to *p* < 0.05, *p* < 0.01, and *p* < 0.001. The final analytical sample size of 1,150 respondents was considered sufficient for stable estimation of key CRS-related proportions within the studied sample and for meaningful subgroup comparisons. In addition, the number of positive top-box outcome events was adequate relative to the number of predictors included in the logistic regression model, supporting reasonably stable coefficient estimation.

## 3 Results of own research

The analysis was based on a survey of *N* = 1,150 respondents. Table 1 summarizes the sample characteristics: women constituted 54.9%, and the sample was predominantly young, with 51.2% aged 18–25 and 29.0% aged 26–35 (overall 80.2% ≤ 35 years). Education was mainly secondary (general 24.6%, technical 28.1%) and higher (25.4%). Most respondents lived in rural areas (37.3%) or towns < 50,000 inhabitants (25.4%), together 62.7% of the sample. Households most commonly reported one or two children (76.1%).

**Table 1:** Demographics of respondents (*N* = 1,150).

Variable	Category	*n*	%
Gender	Female	631	54.9
	Male	519	45.1
Age	18–25	589	51.2
	26–35	333	29.0
	36–45	136	11.8
	46-60	67	5.8
	60 +	25	2.2
Education	Lower secondary	28	2.4
	Vocational	224	19.5
	General secondary	283	24.6
	Technical secondary	323	28.1
	Higher (incl. bachelor and graduate degrees)	292	25.4
Place of residence	Rural	429	37.3
	City/town < 50k	292	25.4
	City 50k−150k	168	14.6
	City 150k−500k	146	12.7
	City >500k	115	10.0
Number of children	1	432	37.6
	2	443	38.5
	3	189	16.4
	More than 3	86	7.5

Table 2 reported self-reported travel frequency with a child in the sample (*N* = 1,150), using five response categories (from “very often” to “very rarely,” plus “don't know”).

**Table 2:** Travel frequency (*N* = 1,150).

Category	*n*	%
Very often	306	26.6
Often	268	23.3
Rarely	323	28.1
Very rarely	167	14.5
Don't know	86	7.5

Nearly half of respondents reported high travel frequency, 26.6% “very often” (*n* = 306) and 23.3% “often” (*n* = 268), totaling 49.9% (*n* = 574). At the same time, 28.1% reported traveling “rarely” (*n* = 323) and 14.5% “very rarely” (*n* = 167), totaling 42.6% (*n* = 490), indicating a substantial group with limited exposure to routine CRS use. A further 7.5% selected “don't know” (*n* = 86), suggesting irregular or seasonal travel patterns. Overall, the distribution indicates heterogeneous exposure to CRS use.

Table 3 presented the self-reported weekly time a child spends in the car seat in the sample (*N* = 1,150), grouped into 4 h categories (up to 1 h, 1–3 h, 3–6 h, and more than 6 h).

**Table 3:** Weekly time spent in the CRS (*N* = 1,150).

Category	*n*	%
Up to 1 h	161	14.0
1–3 h	392	34.1
3–6 h	389	33.8
More than 6 h	208	18.1

Weekly exposure clustered in the middle categories: 34.1% reported 1–3 h (*n* = 392) and 33.8% reported 3–6 h (*n* = 389), totaling 67.9% (*n* = 781) in the 1–6 h range. Lower exposure, up to 1 h, was reported by 14.0% (*n* = 161), while higher exposure, more than 6 h, was reported by 18.1% (*n* = 208). Overall, most respondents reported moderate weekly CRS use, with smaller subgroups at the low- and high-exposure ends, which reflects variation in CRS use.

Table 4 summarized the reported CRS attachment type in the sample (*N* = 1,150), distinguishing seat-belt installation, an ISOFIX base, and a rotating ISOFIX base. ISOFIX-based solutions predominated: 47.9% used an ISOFIX base (*n* = 551) and 26.7% a rotating ISOFIX base (*n* = 307), totaling 74.6% (*n* = 858). Seat-belt installation was reported by 25.4% (*n* = 292).

**Table 4:** Seat attachment type (*N* = 1,150).

Category	*n*	%
Seat belt	292	25.4
ISOFIX base	551	47.9
Rotating ISOFIX base	307	26.7

Table 5 reported the declared CRS installation position in the vehicle (*N* = 1,150): rear seat behind the passenger, rear seat behind the driver, rear-seat middle, and front seat. The most common location was rear seat behind the driver (45.0%, *n* = 517). A substantial proportion reported installation on the front seat (25.4%, *n* = 292) and rear seat behind the passenger (22.4%, *n* = 258), while rear-seat middle was uncommon (7.2%, *n* = 83).

**Table 5:** Seat position in the car (*N* = 1,150).

Category	*n*	%
Rear seat behind passenger	258	22.4
Rear seat behind driver	517	45.0
Rear seat middle	83	7.2
Front seat	292	25.4

Table 6 showed the distribution of reported CRS groups in the sample (*N* = 1,150) by weight/approximate age category (Group 0, 0+, I, II). Group I (9–18 kg) was most common (37.2%, *n* = 428), followed by Group 0 ( ≤ 10 kg) (28.1%, *n* = 323) and Group 0+ ( ≤ 13 kg) (24.3%, *n* = 280). Group II (15–36 kg) was least frequent (10.3%, *n* = 119). Overall, the sample primarily reflects CRS use for infants and younger children, as Groups 0, 0+, and I accounted for 89.6% of responses.

**Table 6:** Seat group (*N* = 1,150).

Category	*n*	%
Group 0: birth to 10 kg	323	28.1
Group 0+: birth to 13 kg	280	24.3
Group I: 9–18 kg	428	37.2
Group II: 15–36 kg	119	10.3

Table 7 summarized the factors reported to influence CRS selection (multi-response, *N* = 1,150). The most frequently cited factors were price (58.1%, *n* = 668), seat weight (48.2%, *n* = 554), and installation method (46.1%, *n* = 530). Social recommendations (39.1%, *n* = 450) and brand (34.0%, *n* = 391) were also common, whereas safety tests (28.3%, *n* = 326) and comfort (26.4%, *n* = 304) were reported less often. Stroller compatibility (13.5%, *n* = 155), design (13.1%, *n* = 151), stability (10.4%, *n* = 120), and material quality (9.1%, *n* = 105) were indicated relatively infrequently.

**Table 7:** CRS selection factors (multi-response; *N* = 1,150).

Driver	*n*	%
Price	668	58.1
Weight	554	48.2
Installation method	530	46.1
Social recommendations	450	39.1
Brand	391	34.0
Safety tests	326	28.3
Comfort	304	26.4
Stroller compatibility	155	13.5
Design	151	13.1
Stability	120	10.4
Material quality	105	9.1

Table 8 presented respondents' stated intention to choose the same CRS again (*N* = 1,150) across five response categories. Positive responses (“yes” or “probably yes”) were reported by 27.4% (*n* = 315), including 15.3% (*n* = 176) “yes” and 12.1% (*n* = 139) “probably yes.” Negative responses (“probably no” or “no”) predominated at 62.3% (*n* = 716), with 37.6% (*n* = 432) selecting “no.” The remaining 10.3% (*n* = 119) chose “difficult to say.”

**Table 8:** Intention to choose the same CRS again (*N* = 1,150).

Category	*n*	%
Yes	176	15.3
Probably yes	139	12.1
Difficult to say	119	10.3
Probably no	284	24.7
No	432	37.6

For the first research question, we examined whether a seller-provided installation demonstration was associated with differences in stated intention to choose the same CRS again. Results are shown in Table 9 (row percentages). Top-box responses (“yes/probably yes”) were very similar when installation was shown (26.8%, *n* = 90/336) vs. not shown (26.2%, *n* = 196/747). The main descriptive difference was a lower share of “probably no” responses in the demonstration group (20.8%) compared with the no-demonstration group (28.0%), while “no” remained common in both groups (40.8% vs. 36.0%). The “uncertain” group (*n* = 67) showed a less stable pattern due to its small size. The association between installation demonstration and intention to choose the same CRS again was statistically significant (χ^2^ = 22.82, *p* = 0.0036), but the effect size was small (Cramér's V = 0.100), indicating a small association.

**Table 9:** Intention to choose the same CRS again by installation demonstration (*n* and row %).

Group	Yes	Probably yes	Difficult to say	Probably no	No
Installation shown (yes)	53 (15.8%)	37 (11.0%)	39 (11.6%)	70 (20.8%)	137 (40.8%)
Installation shown (no)	106 (14.2%)	90 (12.0%)	73 (9.8%)	209 (28.0%)	269 (36.0%)
Uncertain	17 (25.4%)	12 (17.9%)	7 (10.4%)	5 (7.5%)	26 (38.8%)

For the second research question, we tested whether child discomfort during travel (crying/fussiness in the CRS) was associated with a lower intention to choose the same CRS again. For clarity, discomfort responses were collapsed into three groups, frequent discomfort, occasional discomfort, and rare or no discomfort. Results are reported in Table 10. Response distributions were highly similar across discomfort groups. Top-box responses (“yes/probably yes”) were 26.6% in the often group (*n* = 436), 27.8% in the sometimes group (*n* = 388), and 27.9% in the rarely/never group (*n* = 326), with negative responses consistently around two-thirds in each group. The association between discomfort frequency and intention to choose the same CRS again was not statistically significant (χ^2^ = 2.94, *p* = 0.9378), and the effect size was negligible (Cramér's V = 0.036), suggesting no meaningful relationship between this self-reported discomfort indicator and the implementation-relevant outcome in this sample.

**Table 10:** Intention to choose the same CRS again by child discomfort (*n* and row %).

Group	Yes	Probably yes	Difficult to say	Probably no	No
Frequent discomfort	64 (14.7%)	52 (11.9%)	46 (10.6%)	115 (26.4%)	159 (36.5%)
Occasional discomfort	58 (14.9%)	50 (12.9%)	40 (10.3%)	97 (25.0%)	143 (36.9%)
Rare or no discomfort	54 (16.6%)	37 (11.3%)	33 (10.1%)	72 (22.1%)	130 (39.9%)

For the third research question, we assessed whether stronger safety orientation was associated with a higher likelihood of a top-box response (“yes/probably yes”) for choosing the same CRS again. A Safety focus score (range 0–4) was constructed from four components: attention to safety certifications at purchase, citing safety tests as a selection criterion, using ISOFIX attachment, and reporting a system that indicates correct installation. Top-box proportions were then compared across four safety-focus categories. Results are shown in Table 11. Top-box rates were 23.0% in the low category (0–1), 30.0% in the medium category (2), 27.9% in the high category (3), and 30.9% in the very high category (4), noting that the very high group was small (*n* = 55). The association between safety-focus category and top-box response was not statistically significant (χ^2^ = 5.38, *p* = 0.1462), with a small effect size (Cramér's V = 0.068), indicating no clear evidence that higher safety orientation, as operationalized here, translated into stronger implementation-relevant endorsement of the same CRS.

**Table 11:** Top-box intention to choose the same CRS again by safety focus category.

Safety focus category	Top-box (*n*)	Other (*n*)	Top-box %
Low (0–1)	82	274	23.0
Medium (2)	143	334	30.0
High (3)	73	189	27.9
Very high (4)	17	38	30.9

**Figure 1:**
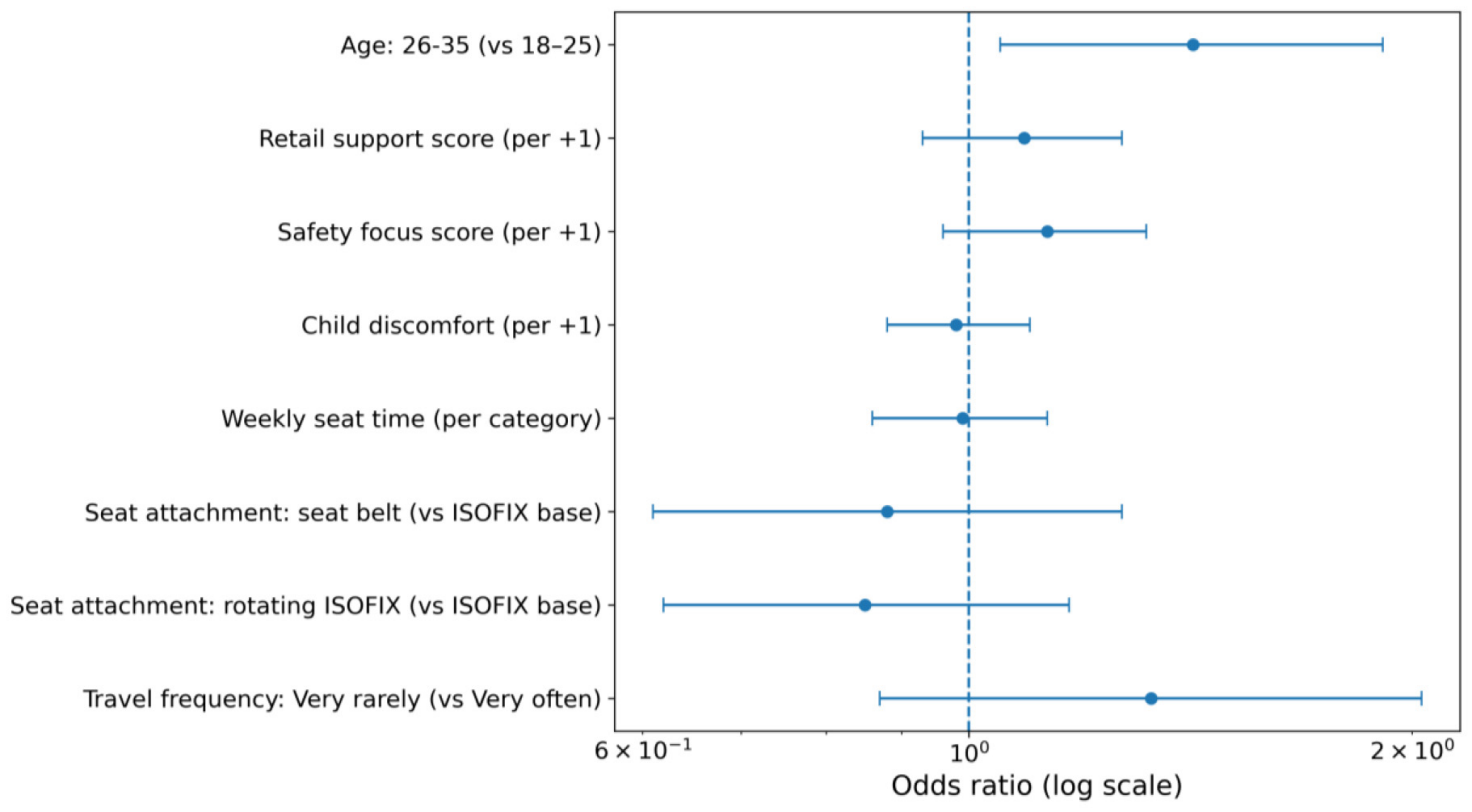
Logistic regression: selected odds ratios (OR) with 95% CI.

For the fourth research question, we used multivariable logistic regression to identify which factors, considered simultaneously, were associated with a top-box response (“yes/probably yes”) for choosing the same CRS again. The dependent variable was dichotomized intention to choose the same CRS again (top-box vs. all other responses). Predictors included Retail support score, Safety focus score, weekly seat time, and child discomfort, with adjustment for travel frequency, attachment type, gender, age, and number of children. Results are reported in Table 12 (OR, 95% CI, *p*), model fit in Table 13, and selected effects in Figure 1.

**Table 12:** Logistic regression: predictors of top-box intention to choose the same CRS again (OR, 95% CI, *p*).

Predictor	OR	CI_low	CI_high	*p*
Travel frequency: very rarely (vs. very often)	1.33	0.87	2.03	0.1851
Travel frequency: often (vs. very often)	1.28	0.89	1.86	0.1886
Travel frequency: rarely (vs. very often)	1.02	0.71	1.47	0.9022
Travel frequency: don't know (vs. very often)	0.84	0.47	1.5	0.5631
Seat attachment: rotating ISOFIX (vs. ISOFIX base)	0.85	0.62	1.17	0.3247
Seat attachment: seat belt (vs. ISOFIX base)	0.88	0.61	1.27	0.4865
Gender: male (vs. female)	1.13	0.87	1.47	0.3674
Age: 26–35 (vs. 18–25)	1.42	1.05	1.91	0.0215
Age: 36–45 (vs. 18–25)	0.9	0.58	1.4	0.6378
Age: 46–60 (vs. 18–25)	0.74	0.39	1.37	0.3345
Age: 60 + (vs. 18–25)	0.69	0.25	1.9	0.4739
Number of children: 2 (vs. 1)	1.17	0.86	1.58	0.3098
Number of children: 3 (vs. 1)	0.98	0.66	1.46	0.9243
Number of children: more than 3 (vs. 1)	1.22	0.73	2.04	0.4582
Retail support score (per +1)	1.09	0.93	1.27	0.2943
Safety focus score (per +1)	1.13	0.96	1.32	0.1511
Child discomfort (per +1)	0.98	0.88	1.1	0.7229
Weekly seat time (per category)	0.99	0.86	1.13	0.8329

**Table 13:** Model fit statistics.

Metric	Value
*N*	1,150.0
Pseudo R^2^ (McFadden)	0.0167
Log-likelihood	−663.9
AIC	1,365.8
BIC	1,461.71

At α = 0.05, most predictors were not statistically significant and had odds ratios close to 1. The only significant association was for age 26–35 years compared with 18–25 years (OR = 1.42, 95% CI 1.05–1.91, *p* = 0.0215). Retail support (OR = 1.09 per point, *p* = 0.2943) and safety focus (OR = 1.13 per point, *p* = 0.1511) showed positive but non-significant trends, while child discomfort (OR = 0.98, *p* = 0.7229) and weekly seat time (OR = 0.99, *p* = 0.8329) were not associated with the outcome. Control variables, including travel frequency, attachment type, gender, and number of children, were also non-significant in this model.

Table 13 indicates limited model explanatory power. The McFadden pseudo-R^2^ was 0.0167, suggesting that the included predictors accounted for only a small proportion of variation in the top-box outcome. Model fit indices were log-likelihood = −663.9, AIC = 1,365.8, and BIC = 1,461.71, consistent with a specification in which no strong predictors of the outcome are captured.

**Figure 2:**
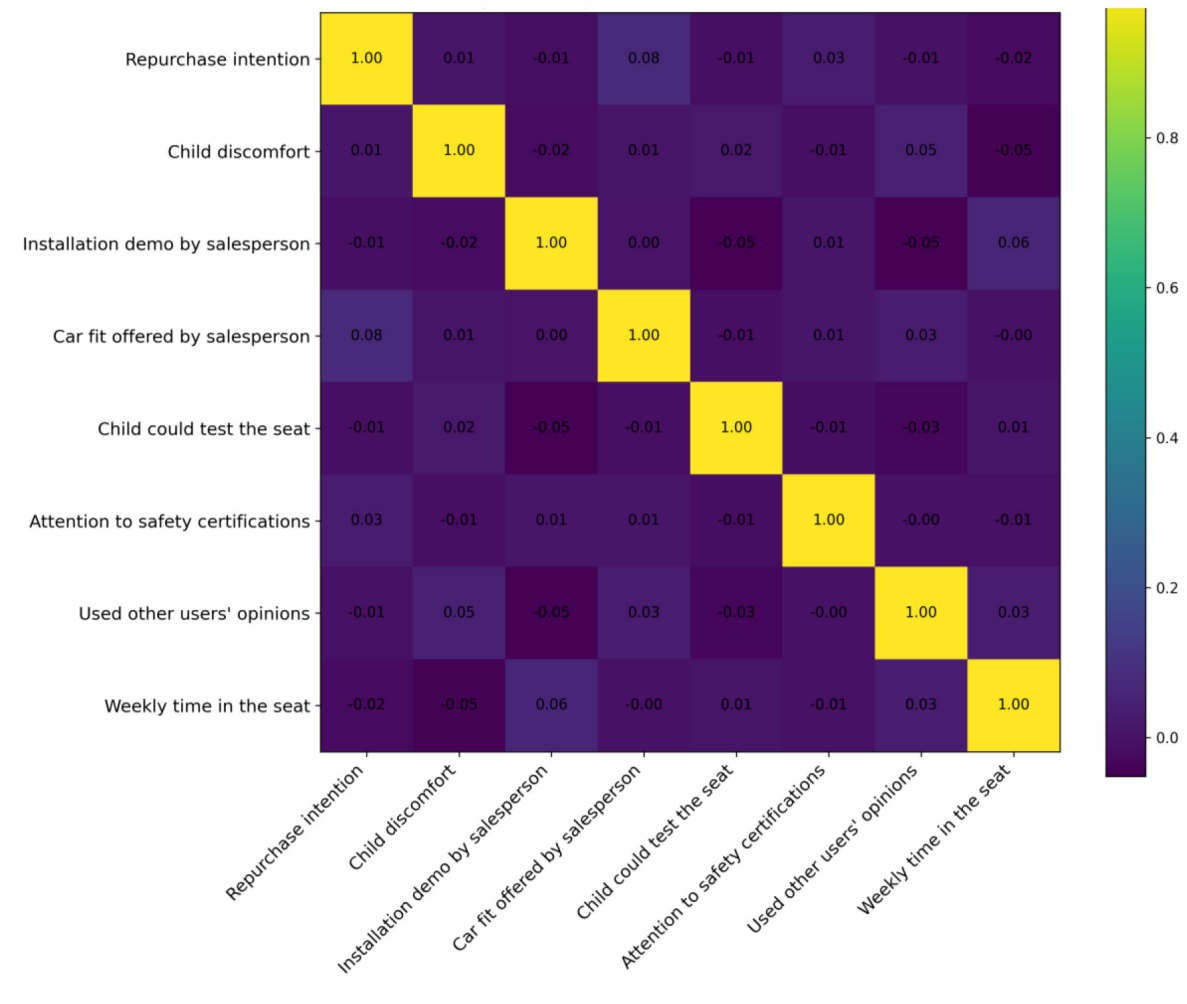
Spearman correlation heatmap (rho).

To provide a compact overview of how responses co-occurred across key variables, we computed a Spearman rank-correlation matrix (ρ) using the following survey items: intention to choose the same CRS again, child crying/fussiness in the CRS, whether the seller demonstrated installation and securing the child, whether the seller offered an in-car fit check, whether the child could test the CRS at purchase, attention to safety certificates, use of other users' advice/opinions, and weekly hours the child spends in the CRS. Results are displayed in Figure 2 as a correlation heatmap. Statistical significance is indicated symbolically in the figure, where a single marker denotes *p* < 0.05, a double marker denotes *p* < 0.01, and a triple marker denotes *p* < 0.001. The largest positive correlation was between intention to choose the same CRS again and reporting that the seller offered an in-car fit check (ρ = 0.08, *p* < 0.05). A second statistically significant, but similarly small, correlation was observed between installation demonstration and weekly CRS use time (ρ = 0.06, *p* < 0.05). Other associations, including the link between child crying/fussiness and intention to choose the same CRS again, were close to zero.

The survey (*N* = 1,150) was dominated by younger caregivers aged ≤ 35 years (80.2%), more often women (54.9%), most commonly from households with 1–2 children (76.1%), and primarily using CRS for the youngest children (Groups 0/0+/I: 89.6%). Exposure to CRS use was moderate, with 49.9% reporting traveling with a child often or very often, 67.9% reporting 1–6 h/week of CRS use, and 18.1% exceeding 6 h/week. ISOFIX-based attachment predominated (74.6%), the most common placement was the rear seat behind the driver (45.0%), and front-seat installation was reported by 25.4%. Reported CRS selection criteria included price (58.1%), seat weight (48.2%), installation method (46.1%), social recommendations (39.1%), brand (34.0%), safety tests (28.3%), and comfort (26.4%). A top-box intention to choose the same CRS again was reported by 27.4% of respondents, while 62.3% reported negative intentions (including 37.6% “no”). In association analyses, seller installation demonstration was significantly associated with the outcome, with a small effect size, whereas child discomfort and safety focus were not statistically significant. In the multivariable logistic model, age 26–35 years (vs. 18–25) was the only significant predictor of a top-box response (OR = 1.42, *p* = 0.0215). The overall model explanatory power was low (McFadden pseudo-R^2^ = 0.0167).

## 4 Discussion

This study adds to the literature on CRS use by showing that caregiver decisions in this sample were shaped primarily by affordability and usability rather than explicitly safety-oriented criteria. Economic and practical considerations appeared to outweigh formal safety indicators in the selection process, while willingness to choose the same CRS again remained limited. This pattern is broadly consistent with previous research showing that CRS-related behavior is influenced not only by safety knowledge, but also by cost, convenience, perceived usability, socioeconomic position, and access to reliable information (22–28). In particular, Moradi et al. and Harzand-Jadidi et al. identified cost, parental knowledge, and socioeconomic conditions as important determinants of CRS use, while Sun et al. emphasized the role of knowledge, motivation, and behavioral skills in caregiver behavior (22–24). Similarly, Sartin et al. and Koppel et al. showed that information environments differ across caregiver groups and may shape whether restraint use is appropriate in practice (25, 26). Al-Awad et al. further synthesized global evidence indicating that barriers to CRS uptake and correct use extend well beyond simple product availability and include legislation, affordability, education, and implementation support (28).

### 4.1 Key findings and resolution of the research problems

The first research question concerned whether seller-provided installation demonstration was associated with intention to choose the same CRS again. The association was statistically significant but small in magnitude, indicating that demonstration alone may have limited influence on caregiver decision-making. This finding is consistent with the intervention literature suggesting that practical instruction can be beneficial, but that one-time or limited support is rarely sufficient to produce substantial behavioral change (13–21). Previous studies indicate that more effective approaches typically involve repeated, guided, or technology-supported assistance rather than a single point-of-sale interaction (17–21). Taken together, these results suggest that installation demonstration is relevant, but likely insufficient as a standalone strategy to substantially improve sustained acceptance or correct-use behavior.

The second research question examined whether child discomfort during travel was associated with intention to choose the same CRS again. No meaningful differences were observed across levels of reported discomfort, suggesting that this factor is not a primary determinant of caregiver satisfaction with the CRS. This aligns with previous findings indicating that barriers to appropriate CRS use are more strongly related to knowledge, confidence, and practical usability than to child discomfort alone (10, 22–24). Although child behavior may influence the overall user experience, it does not appear to function as a key driver of the broader acceptance outcome considered in this study. The third research question addressed whether stronger safety orientation was associated with a higher likelihood of a positive response. Differences across safety-focus categories were modest and not statistically significant, suggesting that declarative safety orientation does not necessarily translate into stronger acceptance of a specific CRS. This is consistent with prior research indicating that while safety knowledge and access to information are important, their behavioral impact depends on clarity, usability, and real-world applicability (23–26). In this context, safety orientation may represent a general attitude rather than a direct predictor of product-related decisions. The fourth research question examined predictors of the outcome in a multivariable framework. Only one age group showed a statistically significant association, while other variables were not significant and overall model explanatory power was low. This suggests that the determinants of sustained acceptance are likely to involve more proximal, experience-based factors not captured in the present survey. Previous research supports this interpretation, highlighting the importance of practical challenges such as installation difficulty, confidence in correct use, and product–vehicle compatibility (10–12, 27). The low explanatory power of the model therefore likely reflects omitted variables related to real-world use rather than the absence of meaningful determinants.

### 4.2 Implementation gap and safety compliance signals

The descriptive profile reinforces the view that CRS use should be understood as a routine behavioral task rather than a one-time purchase decision. A substantial proportion of respondents reported regular exposure to CRS use, indicating repeated opportunities for both correct use and error persistence. From an implementation perspective, this highlights the importance of sustained behavioral support rather than single-point interventions. The attachment profile further illustrates this complexity. Although ISOFIX-based solutions predominated, a considerable proportion of caregivers still relied on seat-belt installation. Previous research shows that misuse remains common even in technically simplified systems, indicating that technological solutions alone are insufficient to ensure correct use (11, 12). This underscores the continued importance of user competence and accessible guidance. Vehicle placement patterns provide an additional signal of implementation challenges. The relatively frequent use of front-seat placement suggests that a substantial subgroup of caregivers may rely on configurations that require greater procedural accuracy. Even without detailed vehicle-level data, this pattern points to the need for clearer, context-specific guidance in real-world settings. The profile of selection determinants provides a particularly clear indication of the implementation gap. Caregiver decisions appear to be shaped primarily by economic and usability considerations, with safety-related criteria playing a secondary role. This is consistent with previous studies showing that real-world decision-making often reflects trade-offs between safety, cost, and convenience (22–28). In practice, caregivers may assume baseline safety compliance and therefore prioritize factors that affect everyday usability. Finally, the observed level of acceptance of the current CRS experience suggests potential challenges for sustained compliance. The relatively low willingness to select the same CRS again may reflect dissatisfaction with the practical demands of use rather than dissatisfaction with safety *per se*. As such, this outcome may serve as an indirect indicator of implementation barriers that are not captured by conventional measures of use versus non-use.

### 4.3 Public health and policy implications

The observed pattern supports several practical implications. First, the association between seller-provided demonstration and the outcome suggests that point-of-sale support has value, but also indicates that this level of intervention is insufficient on its own. This is consistent with evidence showing that more structured, repeated, and skills-based approaches are more effective than isolated information provision (13–21). Second, the combination of diverse installation methods and seat placement patterns suggests that a substantial subgroup of caregivers operates in conditions that are more sensitive to correct-use procedures. This highlights the need for practical demonstration protocols, fit checks, and simplified guidance tailored to real-world transport contexts. Third, the dominance of economic and usability factors in decision-making indicates that public health messaging should move beyond abstract safety appeals and explicitly address affordability and ease of use. Interventions that fail to consider these factors are unlikely to achieve meaningful behavioral change. Finally, the limited acceptance of the current CRS experience, combined with low explanatory power of the model, suggests that caregiver support should extend beyond the point of purchase. The broader literature increasingly emphasizes the value of ongoing, technology-supported guidance, and the present findings reinforce the need for continuous support systems that facilitate correct use over time.

### 4.4 Limitations

Several limitations should be considered when interpreting the findings of this study. First, the study was based on a non-probability convenience sample recruited through social media groups, which implies self-selection of respondents and limits the generalizability of the results beyond the studied sample. Second, all information was self-reported and may therefore be affected by recall error, response bias, or social desirability, particularly in relation to safety-related behaviors. Third, the online format may have favored participation by more digitally active caregivers and may have excluded individuals with limited access to social media or lower engagement with online parenting communities. Finally, the cross-sectional design does not permit causal inference and should be interpreted as describing associations rather than causal relationships. An additional limitation is related to the anonymous online recruitment procedure. Although screening questions were used to assess eligibility, it was not possible to fully verify whether all respondents met the inclusion criteria beyond self-report. Similarly, the study design did not allow complete exclusion of the possibility of multiple submissions by the same participant. However, the survey did not include financial or material incentives, which reduces the likelihood of intentional repeated participation, and the large sample size limits the potential impact of such occurrences on the overall findings.

## 5 Summary and conclusions

This large cross-sectional survey of caregivers (N = 1,150) provides an implementation-focused profile of CRS-related behaviors and highlights a persistent implementation gap between safety recommendations and everyday CRS practice. The sample was dominated by caregivers of younger children (Groups 0/0+/I: 89.6%) with frequent exposure to child transport, with 49.9% reporting travel with a child often or very often and 67.9% reporting 1–6 h per week of CRS use. These exposure patterns matter because they increase the number of opportunities for both correct use and error persistence, making sustained adherence a central determinant of injury risk mitigation.

Several findings have direct safety compliance relevance. Although ISOFIX-based attachment was common (74.6%), a substantial minority reported seat-belt installation (25.4%), indicating ongoing need for practical competence in belt routing and tightening. Importantly, 25.4% reported installing the CRS on the front seat, a configuration that is particularly sensitive to vehicle-specific procedures and therefore may carry heightened risk if correct-use steps are not consistently followed. Reported selection criteria were dominated by feasibility constraints, price (58.1%), seat weight (48.2%), and installation method (46.1%), while explicitly safety-referenced evidence, such as safety tests, was endorsed less frequently (28.3%). This pattern suggests that, in real-world settings, economic and usability considerations may shape decisions and routine behaviors more strongly than safety signals alone, reinforcing the need for implementation strategies that reduce practical burden and support correct use.

The declared willingness to choose the same CRS again was low (27.4% top-box), while negative intentions predominated (62.3%, including 37.6% “no”). Interpreted through an implementation lens, this outcome can be viewed as a potential marker of dissatisfaction that may undermine sustained safety compliance, through reduced motivation to maintain correct use, switching products without structured guidance, or acceptance of suboptimal configurations perceived as easier. Consistent with this interpretation, seller installation demonstration showed a statistically detectable but small association with the outcome, whereas child discomfort and safety focus did not robustly differentiate responses, and multivariable modeling identified only the 26–35 age group as a significant predictor with very low overall explanatory power (McFadden pseudo-R^2^ = 0.0167). Together, these results indicate that key determinants of sustained adherence may lie in more proximal, experience-based factors not captured in the survey, such as recurring installation difficulties, vehicle–CRS compatibility constraints, and the adequacy of post-purchase support for maintaining correct use as the child grows.

From a public health perspective, the findings support implementation-oriented action aligned with Sustainable Development Goal (SDG) 3.6, which targets the reduction of road traffic deaths and injuries. Priorities include standardizing point-of-sale education, ensuring routine installation demonstration accompanied by basic competency checks, and reinforcing correct use through public campaigns and scalable technology-enabled supports. Given the large share of respondents from rural areas and small towns, findings may reflect differential access to safety education in rural vs. urban settings, strengthening the case for equitable, accessible implementation strategies that can reach caregivers outside major urban centers. Improving CRS safety is not solely a matter of product availability but of behavioral adherence, structured education, and implementation-focused public health policy.

## Data Availability

The raw data supporting the conclusions of this article will be made available by the authors, without undue reservation.
